# Gastric acid inhibitor aggravates indomethacin-induced small intestinal injury via reducing *Lactobacillus johnsonii*

**DOI:** 10.1038/s41598-019-53559-7

**Published:** 2019-11-25

**Authors:** Yuji Nadatani, Toshio Watanabe, Wataru Suda, Akinobu Nakata, Yuji Matsumoto, Satoshi Kosaka, Akira Higashimori, Koji Otani, Shuhei Hosomi, Fumio Tanaka, Yasuaki Nagami, Noriko Kamata, Koichi Taira, Hirokazu Yamagami, Tetsuya Tanigawa, Masahira Hattori, Yasuhiro Fujiwara

**Affiliations:** 1 Department of Gastroenterology, Osaka City University Graduate School of Medicine, Osaka, Japan; 2 Laboratory for Microbiome Sciences, Center for Integrative Medical Sciences, RIKEN, Kanagawa, Japan; 3 Graduat Graduate e School of Advanced Science and Engineering, Waseda University, Tokyo, Japan

**Keywords:** Gastrointestinal diseases, Gastrointestinal diseases, Inflammation, Inflammation

## Abstract

Proton pump inhibitors (PPIs) alter the composition of the intestinal microbiome, exacerbating indomethacin (IND)-induced small intestinal damage. Vonoprazan fumarate inhibits gastric acid secretion using a different mechanism from PPIs. We investigated the effects of both drugs on the intestinal microbiome and IND-induced small intestinal damage. We sought to clarify whether PPI-induced dysbiosis and worsening of the damage were due to a specific drug class effect of PPIs. Rabeprazole administration increased operational taxonomic unit numbers in the small intestines of C57BL/6 J mice, whereas the difference was not significant in the vonoprazan-treated group but exhibited a trend. Permutational multivariate analysis of variance of the unweighted UniFrac distances showed significant differences between vehicle- and vonoprazan- or rabeprazole-treated groups. *L. johnsonii* was the predominant microbial species, and the population ratio decreased after vonoprazan and rabeprazole administration. The vonoprazan- and rabeprazole-treated groups showed increased IND-induced damage. This high sensitivity to IND-induced damage was evaluated by transplantation with contents from the small intestine of mice treated with either vonoprazan or rabeprazole. Supplementation of *L. johnsonii* orally in mice treated with rabeprazole and vonoprazan prevented the increase in IND-induced small intestinal damage. In conclusion, both rabeprazole and vonoprazan aggravated NSAID-induced small intestinal injury by reducing the population of *L. johnsonii* in the small intestine via suppressing gastric acid secretion.

## Introduction

Proton pump inhibitors (PPIs) are commonly used to treat peptic ulcer, gastro esophageal reflux disease (GERD), and eosinophilic esophagitis, to name a few. Although PPIs are very effective in treating the appropriate indications, long-term PPI use often results in adverse events including bone fracture^1^, pneumonia^2^, and *Clostridium difficile* infection^3^. Additionally, PPI raises the pH in the stomach, altering the gastric microbiome. Gastric acid suppression by PPIs also displays a downstream effect on small intestinal bacterial composition^4^.

Small intestinal injury is one of the major adverse effects experienced by patients who receive non-steroidal anti-inflammatory drugs (NSAIDs) to reduce inflammation such as joint pain, stiffness, and swelling. Recent studies using video capsule endoscopy or double balloon endoscopy detected small intestinal mucosal breaks such as ulcers and erosions in most of the chronic users of NSAIDs^5,6^. Unlike gastric damage where gastric acid is a major player, NSAID-induced small intestinal injury is independent of gastric acid^7^. Therefore, PPIs cannot prevent such injury^8^. On the contrary, many reports have suggested that PPIs exacerbate NSAID-induced small intestinal injury^9,10^. One of the considered causes of injury deterioration due to PPI administration is an imbalance in the intestinal microbiome^11^, because the intestinal microbiome has been proven to play a crucial role in the pathogenesis of the injury. The ulcerogenic effect to the small intestine by NSAIDs was markedly reduced in germ-free rats or mice given broad-spectrum rats, while lipopolysaccharide (LPS), a major component of the outer membrane of gram-negative bacteria acted as a complicating factor in the injury^12–15^. However, nowadays, there is limited information on PPI-induced intestinal dysbiosis and its underling mechanisms.

Recently, vonoprazan fumarate, a novel, active potassium-competitive acid blocker (P-CAB), was launched in Japan before entering the world market. Vonoprazan exhibits a stronger inhibitory effect on gastric acid secretion than traditional PPIs such as rabeprazole and esomeprazole in humans^16^. Presently, vonoprazan fumarate is widely prescribed as a maintenance therapy for GERD, eradication of *Helicobacter pylori*, and treatment of gastroduodenal ulcer in Japan^17,18^.

In the present study, we investigated the effects of rabeprazole on the intestinal microbiome using a next generation sequencing (NGS) and mice with indomethacin-induced small intestinal damage. Similar experiments were also performed in mice given vonoprazan, which suppresses gastric acid secretion by a different mechanism from PPIs. This was performed to clarify whether PPI-induced deterioration of the damage and dysbiosis were due to the acid suppressive effect or a specific drug class effect of PPIs.

## Materials and Methods

### Animals

Wild-type C57BL/6 mice were purchased from Charles River Japan Inc. (Atsugi, Japan), and specific pathogen-free 7-week-old male mice were used in the study similar to that in our previous study^14,19^. All animals were housed in polycarbonate cages with paper chip bedding. The cages were located in an air-conditioned biohazard room under a 12-h light-dark cycle.

All experiments were carried out under the control of animal research committee in accordance with the Guidelines on Animal Experiments in Osaka City University Graduate School of Medicine, the Japanese Government Animal Protection and Management Law (No. 105), and the Japanese Government Notification on Feeding and Safekeeping of Animals (No. 6). All experimental procedures were approved by the Animal Care Committee of Osaka City University Graduate School of Medicine (Approval number 16008). All surgeries were performed under isoflurane, with maximal efforts taken to minimize suffering.

### Intraperitoneal injection of rabeprazole and vonoprazan

To investigate the effect of PPIs or a P-CAB on the small intestinal microbiome, mice were administered intraperitoneal injections of rabeprazole sodium (0–20 mg/kg; MilliporeSigma Co., St. Louis, MO), vonoprazan fumarate (0–20 mg/kg; Chemscene, Monmouth Junction, NJ), or vehicle (saline only), for seven days. Mice were euthanized 24 h after the final intraperitoneal injection.

### Sample collection and microbiome analysis

The lower small intestinal or cecum contents were collected immediately after sacrificing, using a feces collection kit (Takara bio, Shiga, Japan). Genomic DNA was then isolated using the NucleoSpin Microbial DNA Kit (MACHEREY-NAGEL, Düren, Germany). Approximately 500 µL of stored fecal samples were placed in a microcentrifuge tube containing 100 µl Elution Buffer BE. The mixture was then placed into the NucleoSpin Beads Tube with proteinase K, which was subjected to mechanical beads-beating for 12 min at 30 Hz in the TissueLyzer LT. The subsequent extraction procedure was performed per the manufacturer’s instructions. Extracted DNA samples were purified using the Agencourt AMPure XP (Beckman Coulter, Brea, CA).

Two-step PCRs were performed for the purified DNA samples to obtain sequence libraries. The first PCR was performed using a 16S (V3–V4) Metagenomic Library Construction Kit for NGS (Takara Bio Inc., Kusatsu, Japan) with the primer pairs: 341 F, 5′-TCGTCGGCAGCG TCAGATGTGTATAAGAGACAGCCTACGGGNGGCWGCAG-3′; and 806 R, 5′-GTCTCGTGGGCTCGGAGATGTGTATAAGAG ACAGGGACTACHVGGGTWTCTAAT-3′ which correspond to the V3–V4 region of the 16S rRNA gene. The second PCR was performed to add the index sequences for Illumina sequencer with barcode sequence using the Nextera XT Index kit (Illumina, San Diego, CA). The prepared libraries were subjected to sequencing of paired-end 300 bases using the MiSeq Reagent Kit v3 on the MiSeq (Illumina) at the Biomedical Center, Takara Bio.

### Data processing of 16S rRNA sequences

After demultiplexing of 16S sequence reads, paired-end reads were joined using the fastq-join program as in previous studies^20^. Reads with an average quality value < 25 and inexact matches to both universal primers, which totally accounted for 43–44% of all reads, were removed. Among the filter-passed reads, 3,000 reads per sample were randomly chosen and used for the analysis.

We then sorted the selected reads by the average quality value and grouped them into OTUs by clustering using the UCLUST algorithm with a 97% identity threshold. Taxonomic assignments for each OTU were made by similarity searching against the publicly available 16S (RDP and CORE), and NCBI genome databases using the GLSEARCH program. For assignment at the phylum, family, genus, and species levels, sequence similarity thresholds of 70%, 90%, 94% and 96% were respectively applied^20^.

### Transplantation of fecal microbiota from small intestinal lumen contents

For fecal microbiota transfer of fresh small intestinal lumen contents, recipient mice received ampicillin (1 g/L; Wako, Osaka, Japan), neomycin (1 g/L; Wako), metronidazole (500 mg/L; Wako) and vancomycin (500 mg/L; Wako) in drinking water for 14 consecutive days, while donor mice received rabeprazole or vonoprazan intraperitoneally for 7 days. One day before fecal microbiota transfer, drinking water with antibiotic cocktail was removed, and the mice fed ordinary drinking water. For five consecutive days, small intestinal contents of donor mice were orally administered, and small intestinal microbiota was transplanted in the recipient mice.

### Calculation of bacterial number in the small intestinal or colon luminal contents

The total number of a wide variety of bacterial strains was measured by bacteria (tuf gene) quantitative PCR kit (Takara Bio.), according to the manufacturer’s manual. Briefly, fecal DNA was extracted using the Nucleospin Tissue kit (Takara Bio.) according to the manufacturer’s manual. Expression of tuf gene was measured by SYBR green real-time PCR using an Applied Biosystems 7500 Fast Real-Time PCR system and software (Life Technologies Corporation). The bacterial copy number per gram of specimen was calculated from copy number of tuf gene by the standard curve method.

### Culture and administration of *lactobacillus johnsonii (L. johnsonii), L. intestinalis*, *and L. marinus*

*L. johnsonii* and *L. marinus* were obtained from NITE (Osaka, Japan). *L. intestinalis* was obtained from the American Type Culture Collection (Virginia USA). *L. johnsonii*, *L. intestinalis*, and *L. marinus* were incubated in MRS broth (MilliporeSigma) or MRS broth with agar, according to the manufacturer’s protocol. *L. johnsonii*, *L. intestinalis*, and *L. marinus* (10^5^-10^6^ CFU /mouse) were administered by gavage for 7 days, and animals were sacrificed 24 h later.

### Experimental induction of small intestinal injury by indomethacin

To induce small intestinal injury, we administered 10 mg/kg of indomethacin (IND) (MilliporeSigma Co.) in a 0.5% carboxymethylcellulose solution (Wako, Osaka, Japan) by gavage to non-fasting animals^14,19^. Animals were then sacrificed 24 h later. In each case, to evaluate the tissue damage, 1% Evans blue (Wako) was injected intravenously (i.v.) 30 min before sacrifice, and the small intestine opened along the antimesenteric attachment. The areas (mm^2^) of the macroscopically visible lesions were measured, summed per small intestine, and used as the lesion index. All animals had free access to food and water during the experiments.

### RNA isolation and determination of mRNA expression levels of the inflammatory cytokines using RT-PCR

The mRNA expression levels of inflammatory cytokines were measured with the same methods employed in our previous studies using RT-PCR^14,19^. Total RNA was isolated from small intestinal tissue using an ISOGEN kit (Nippon Gene Co., Ltd., Tokyo, Japan) according to the manufacturer’s protocol. Complementary DNA was produced using the High Capacity RNA-to-cDNA Kit (Life Technologies Corporation, Carlsbad, CA) according to the manufacturer’s protocol. Real-time quantitative RT-PCR analyses were performed using an Applied Biosystems 7500 Fast Real-Time PCR system and software (Life Technologies Corporation); the reaction mixture was prepared according to the manufacturer’s protocol using the TaqMan Fast Universal PCR master mixture (Life Technologies Corporation). The PCR thermal cycling conditions were: 45 cycles at 95 °C for 15 s and 60 °C for 1 min. The expression levels of interleukin (IL)-1β and tumor necrosis factor (TNF)-α in the small intestine and colon tissue were quantified using real-time RT-PCR, and standardized to TaqMan glyceraldehyde-3-phosphate dehydrogenase (GAPDH; Life Technologies Corporation) mRNA levels. The expression levels of these mRNAs are indicated as ratios to the mean value of the vehicle-treated intestinal tissue. The primers and probes used for RT-PCR are detailed in Supplemental Table 1.

### Measurement of serum gastrin levels

Blood samples were collected from mice after rabeprazole or vonoprazan administration for 7 days, and serum gastric levels were measured by a radioimmunoassay using the polyethylene glycol method.

### Intraperitoneal injection of gastrin

To investigate the effect of hypergastrinemia on NSAID-induced small intestinal injury, mice were administered an intraperitoneal injection of gastrin (1 or 10 μg/mouse; Backem AG, Bubendorf, switzerland) or vehicle for 7 days. At the same time as the last intraperitoneal injection, 10 mg/kg of IND was administered orally as described previously herein. Mice were euthanized 24 h after the final intraperitoneal injection.

### Statistical analysis

Values are expressed as means ± SEM. Significant differences among treatment group means were found using one-way analysis of variance, and the results were analyzed by Fisher’s protected least-significant-difference test using SPSS version 21. To reveal differences between groups, we performed PERMANOVA (“adonis” command, R-package vegan) based on UniFrac distance. A p value < 0.05 was considered significant.

## Results

### Rabeprazole and vonoprazan alter the gastrointestinal microbiome with elevated serum gastrin levels

**Figure 1:**
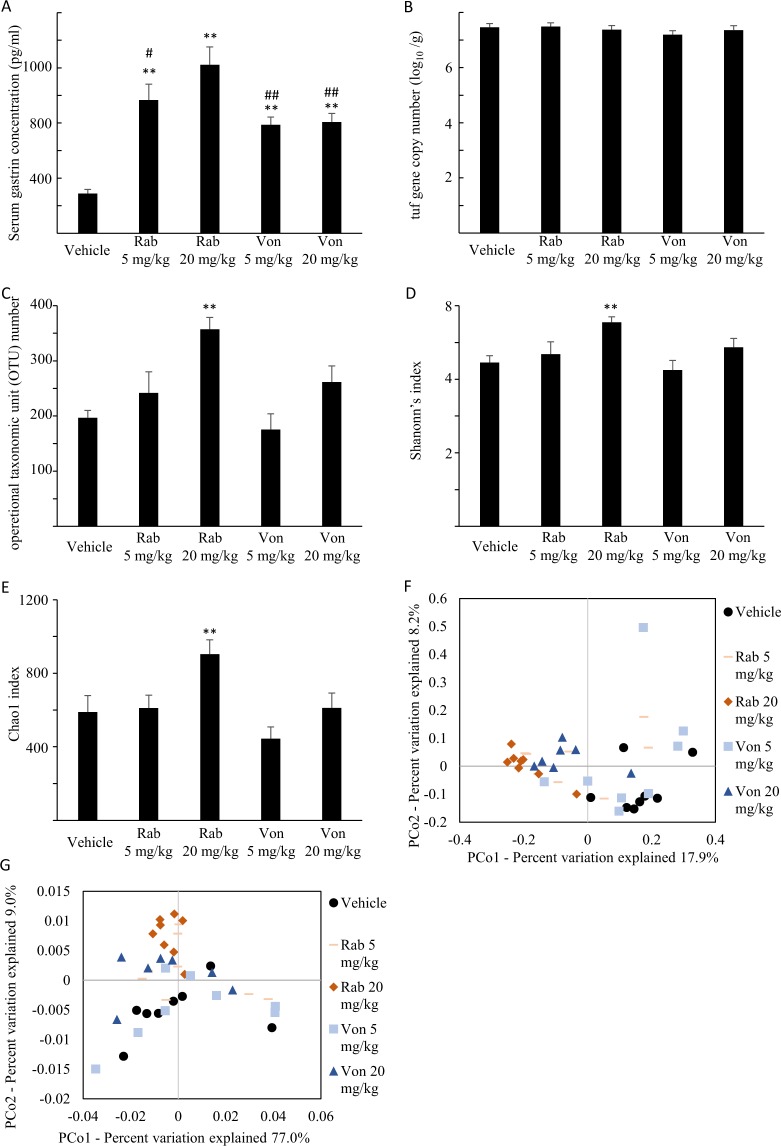
Changes in the small intestine microbiome and serum gastrin levels after administration of rabeprazole and vonoprazan. (**A**) Serum gastrin concentration in mice given rabeprazole (Rab), vonoprazan (Von) or vehicle. Serum gastric concentration was measured by a radioimmunoassay using the polyethylene glycol method, N = 7–8. **P < 0.01 compared to vehicle-treated mice. ^#^P < 0.05, ^##^P < 0.01 compared to Rab 20 mg/kg administered group. (**B**) Total number of a wide variety of small intestinal bacterial strains in mice given Rab, Von or vehicle. Total number of bacterial strains were calculated based on the tuf gene copy number per gram of luminal contents of small intestine, N = 7–8. (**C**–**E**) Alpha-diversity, measured by Operational taxonomic unit (OTU) number (**C**), Shannon’s diversity index (**D**), and Chao1 index (**E**) of small intestinal lumen contents in mice given Rab, Von or vehicle, N = 7–8. *P < 0.05 compared to the OTU number, Shannon’s diversity index, and Chao1 index in vehicle-treated mice, respectively. (**F**,**G**) Principal coordinate analysis (PCoA) of unweighted (**F**) and weighted (**G**) UniFrac distances of small intestinal lumen contents.

Serum concentration of gastrin, which reflects the extent of the inhibition of gastric acid secretion, was markedly elevated by the administration of low and high doses of rabeprazole and vonoprazan (Fig. 1A). There were significant differences in serum concentrations of gastrin between the 20 mg/kg rabeprazole-treated group and the 20 mg/ kg vonoprazan-treated group.

A significant difference was not found in the tuf gene copy number, which reflects number of bacteria, in the small intestine between the drug administered groups and vehicle group (Fig. 1B). Rabeprazole administration increased OTU number, and vonoprazan administration tended to increase the OTU (p = 0.110) of the small intestinal microbiome (Fig. 1C). Shannon’s index and Chao index were also elevated by the administration of rabeprazole (Fig. 1D,E). Permutational multivariate analysis of variance (PERMANOVA) of the unweighted UniFrac distances showed significant differences between the vehicle- and vonoprazan- or rabeprazole-treated groups in the small intestine, while PERMANOVA of the weighted UniFrac distances showed significant differences between the vehicle- and rabeprazole (20 mg/kg)-treated groups (Fig. 1F,G and Table 1). These results suggest that rabeprazole and vonoprazan administration altered the small intestinal microbiome.

**Table 1:** PERMANOVA of unifrac distance in small intestine.

Category	No. subject	Weigthed UniFrac		Unweigthed UniFrac	
		R^2^	P value	R^2^	P value
vehicle vs rabeprazole 5 mg/kg	vehicle:8 rabeprazole 5 mg/kg:8	0.09	0.26	0.13	0.01
vehicle vs rabeprazole 20 mg/kg	vehicle:8 rabeprazole 20 mg/kg:8	0.26	<0.01	0.23	<0.01
vehicle vs vonoprazan 5 mg/kg	vehicle:8 vonoprazan 5 mg/kg:8	0.07	0.35	0.10	0.04
vehicle vs vonoprazan 20 mg/kg	vehicle:8 vonoprazan 20 mg/kg:7	0.04	0.50	0.17	<0.01
rabeprazole 5 mg/kg vs vonoprazan 5 mg/kg	rabeprazole 5 mg/kg:8 vonoprazan 5 mg/kg:8	0.04	0.51	0.10	0.04
rabeprazole 20 mg/kg vs vonoprazan 20 mg/kg	rabeprazole 20 mg/kg:8 vonoprazan 20 mg/kg:7	0.08	0.31	0.13	<0.01

The relative abundance of the dominant phyla, genus and species in the small intestine is presented in Tables 2–4, respectively. At the phylum level, members of Firmicutes, Bacteroidetes, Verrucomicrobia, Actinobacteria, Proteobacteria, Deferribacteres and Tenericutes were identified. Actinobacteria were significantly decreased by the administration of rabeprazole at a high dose, and vonoprazan (Table 2 and Supplemental Table 6). The difference in the composition of genus between vehicle-treated and drug-treated mice is shown in Table 3 and Supplemental Table 7. Members of *Lactobacillus* were the most dominant genus in the small intestine, but a significant difference was not found between vehicle-treated and drug-treated groups (Table 3 and Supplemental Table 7). At the species level, the most dominant species was *L. johnsonii*. Rabeprazole administration decreased the abundance ratio of *L. johnsonii* in the small intestine, while the administration of vonoprazan tended to decrease the abundance ratio (p = 0.057) (Table 4 and Supplemental Table 8). Classified as the same genus as *L. johnsonii*, the population of *L. intestinalis* was increased by low and high doses of rabeprazole and low doses of vonoprazan. The population of *L. murinus* was also elevated with low-dose rabeprazole and high-dose vonoprazan in the small intestine (Table 4).

**Table 2:** The major bacterial composition of small intestine at phylum level. (%).

	vehicle	rabeprazole		vonoprazan	
		5 mg/kg	20 mg/kg	5 mg/kg	20 mg/kg
Firmicutes	51.14 ± 7.35	59.55 ± 6.43	56.13 ± 1.54	54.63 ± 9.43	50.51 ± 7.47
Bacteroidetes	46.60 ± 7.42	37.17 ± 6.03	41.28 ± 1.40	42.45 ± 9.38	44.68 ± 5.85
Verrucomicrobia	0.30 ± 0.06	1.78 ± 0.63*	1.65 ± 0.28*	1.23 ± 0.53	1.27 ± 0.58
Actinobacteria	1.64 ± 0.16	1.43 ± 0.24	0.89 ± 0.12**	0.58 ± 0.14**	0.78 ± 0.22**
Proteobacteria	0.32 ± 0.14	0.05 ± 0.04	0.00 ± 0.00	0.99 ± 0.44	2.74 ± 2.71

Identity ≥70% Mean ± s.e. *p < 0.05 vs Vehicle, **p < 0.01 vs Vehicle.

**Table 3:** The top 10 major bacterial composition of small intestine at genus level. (%).

	vehicle	rabeprazole		vonoprazan	
		5 mg/kg	20 mg/kg	5 mg/kg	20 mg/kg
Lactobacillus	37.56 ± 5.66	46.71 ± 7.74	33.77 ± 2.59	45.45 ± 7.56	35.46 ± 7.92
Clostridium	4.12 ± 1.12	5.34 ± 1.72	9.73 ± 1.45*	1.91 ± 0.49	7.94 ± 0.89*
Akkermansia	0.30 ± 0.06	1.76 ± 0.62*	1.60 ± 0.26*	1.21 ± 0.53	1.22 ± 0.56
Eubacterium	0.46 ± 0.28	0.42 ± 0.11	0.43 ± 0.09	0.17 ± 0.05	0.43 ± 0.06
Roseburia	0.02 ± 0.01	0.19 ± 0.06	0.31 ± 0.09*	0.17 ± 0.12	0.23 ± 0.08
Candidatus Arthromitus	2.94 ± 0.89	0.89 ± 0.60	0.33 ± 0.16*	2.34 ± 1.48	0.50 ± 0.33
Ruminococcus	0.03 ± 0.02	0.18 ± 0.11	1.10 ± 0.27**	0.15 ± 0.09	0.31 ± 0.15
Oscillospira	0.01 ± 0.01	0.38 ± 0.17*	0.53 ± 0.09*	0.03 ± 0.02	0.53 ± 0.17*
Parabacteroides	0.01 ± 0.01	0.13 ± 0.06	0.42 ± 0.08*	0.09 ± 0.07	0.35 ± 0.07*
Escherichia	0.23 ± 0.13	0.05 ± 0.03	0.00 ± 0.00	0.97 ± 0.44	2.51 ± 2.49

Identity ≥94% Mean ± s.e. *p < 0.05 vs Vehicle, **p < 0.01 vs Vehicle.

**Table 4:** The top 20 major bacterial composition of small intestine at species level. (%).

			vehicle	rabeprazole		vonoprazan	
		% id		5 mg/kg	20 mg/kg	5 mg/kg	20 mg/kg
total_OTU00007	Lactobacillus johnsonii	100.0	18.20 ± 4.24	12.26 ± 2.00	8.43 ± 1.21*	12.77 ± 3.45	10.03 ± 2.40^+^
total_OTU00029	Barnesiella intestinihominis	86.5	13.34 ± 3.10	11.34 ± 2.08	11.33 ± 1.29	9.63 ± 2.19	13.90 ± 1.29
total_OTU00016	Lactobacillus intestinalis	100.0	5.59 ± 1.00	11.54 ± 2.31*	11.40 ± 0.82*	17.61 ± 3.01**	8.11 ± 1.60
total_OTU00013	Lactobacillus reuteri	100.0	8.63 ± 1.29	11.95 ± 2.79	6.98 ± 0.66	8.26 ± 1.64	7.75 ± 1.96
total_OTU00041	Barnesiella intestinihominis	88.6	7.69 ± 1.94	5.90 ± 0.95	6.75 ± 0.66	6.73 ± 1.57	5.63 ± 0.85
total_OTU00024	Lactobacillus murinus	100.0	3.11 ± 1.04	8.44 ± 1.32*	4.41 ± 0.52	5.06 ± 1.42	7.80 ± 2.93*
total_OTU00096	Barnesiella intestinihominis	86.9	4.05 ± 0.70	3.63 ± 0.70	3.96 ± 0.31	4.75 ± 0.73	5.09 ± 0.73
total_OTU00129	Porphyromonas HF001	86.0	3.49 ± 0.68	3.28 ± 0.61	2.53 ± 0.34	2.68 ± 0.63	3.48 ± 0.52
total_OTU00116	Porphyromonas catoniae	88.1	2.37 ± 0.48	1.99 ± 0.46	3.03 ± 0.29	3.63 ± 0.91	3.28 ± 0.60
total_OTU00097	Porphyromonas catoniae	86.0	2.40 ± 0.46	2.20 ± 0.37	1.86 ± 0.17	3.28 ± 0.82	2.42 ± 0.56
total_OTU00188	Barnesiella intestinihominis	87.2	1.40 ± 0.34	1.65 ± 0.29	2.20 ± 0.22	1.68 ± 0.44	1.74 ± 0.43
total_OTU00546	Porphyromonas catoniae	86.9	3.54 ± 0.69	0.86 ± 0.16**	1.13 ± 0.09**	1.98 ± 0.47**	1.04 ± 0.24**
total_OTU00540	Porphyromonas catoniae ATCC 51270	85.0	1.62 ± 0.31	1.46 ± 0.37	1.49 ± 0.15	1.72 ± 0.43	1.90 ± 0.50
total_OTU00017	Candidatus Arthromitus sp. SFB-mouse	100.0	2.86 ± 0.86	0.86 ± 0.59	0.31 ± 0.15*	2.33 ± 1.48	0.49 ± 0.32
total_OTU00208	Akkermansia muciniphila	100.0	0.30 ± 0.06	1.74 ± 0.61*	1.56 ± 0.26	1.18 ± 0.52	1.21 ± 0.56
total_OTU00207	Bacteroides sp. Tilapia9	84.1	1.04 ± 0.20	1.04 ± 0.30	1.17 ± 0.14	1.04 ± 0.25	1.08 ± 0.27
total_OTU00127	Clostridium disporicum	99.5	1.75 ± 0.46	0.06 ± 0.03*	0.01 ± 0.01*	0.81 ± 0.27	2.21 ± 1.12
total_OTU00276	Eubacterium biforme DSM 3989	87.3	0.71 ± 0.19	0.72 ± 0.16	2.36 ± 0.26**	0.00 ± 0.00**	0.07 ± 0.02*
total_OTU00399	Escherichia coli	100.0	0.23 ± 0.13	0.05 ± 0.03	0.00 ± 0.00	0.94 ± 0.42	2.49 ± 2.47
total_OTU00143	[Bacillus] sp. KITNT-3	100.0	1.79 ± 1.20	0.05 ± 0.03*	0.00 ± 0.00*	0.84 ± 0.48	0.11 ± 0.07

Mean ± s.e. *p < 0.05 vs Vehicle, **p < 0.01 vs Vehicle, ^+^ P = 0.059.

Colon samples from the same mice were examined as the small intestine sample. There was no significant difference in the number of tuf gene copy, OTU number and Chao1 index of colon between the drug administered groups and vehicle group (Supplemental Fig. 1A–D). PERMANOVA of the unweighted UniFrac distances showed significant differences between the vehicle- and vonoprazan- or rabeprazole-treated groups, while PERMANOVA of the weighted UniFrac distances showed significant differences between the vehicle- and rabeprazole-treated groups (Supplemental Fig. 1E,F and Table 5). The relative abundance of the dominant phyla, genus and species in cecum contents is presented in Supplemental Tables 2–4, respectively. In colon, Firmicutes was the most dominant phylum and Bacteroidetes was the second dominant phylum (Supplemental Tables 2 and 6). At the genus level, Lactobacillus was the second dominant phylum, and its population was significantly elevated by the treatment of any doses of rabeprazole and vonoprazan (Supplemental Tables 2 and 7). At the species level, the major species in colon were slightly different from those in the small intestine. *L. johnsonii* was the third dominant species, and its population did not decrease by treatment with rabeprazole and vonoprazan, unlike the results obtained for the small intestine (Supplemental Tables 4 and 8).

### Rabeprazole and vonoprazan exacerbate IND-induced small intestinal injury

**Figure 2:**
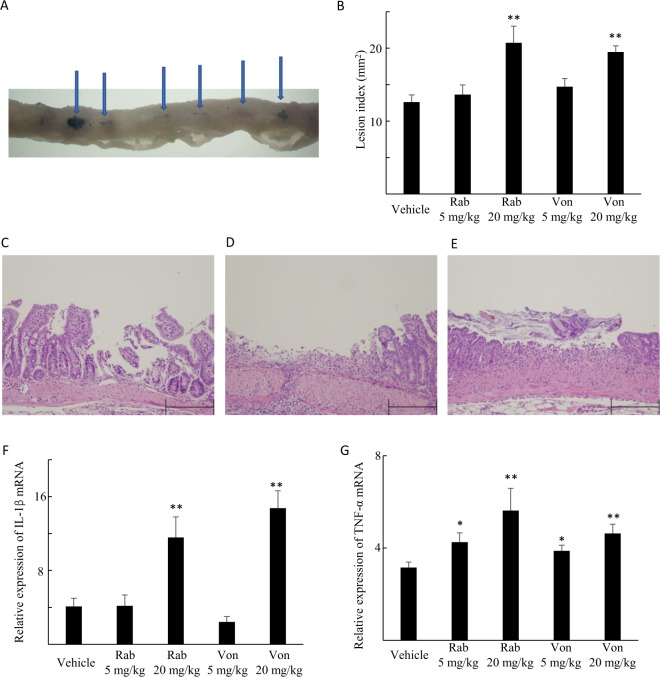
Small intestinal damage and cytokine expression after indomethacin administration to rabeprazole- (Rab)-, vonoprazan- (Von)- and vehicle-treated mice. (**A**) Mucosa injured (arrows) by indomethacin and stained dark blue using 1% Evans blue. (**B**) The effect of Rab and Von on indomethacin-induced small intestinal injury. The areas of the macroscopically visible lesions, stained with Evans blue, were measured, summed per small intestine, and used as the lesion index. Each column represents the mean ± standard error of the mean (SEM). **P < 0.01, *P < 0.05 vs. vehicle-treated controls, N = 7–8. (**C**–**E**) Histological findings of indomethacin-induced small intestinal injury in vehicle- (**C**), Rab- (**D**) or Von- (**E**) treated group. Indomethacin caused small intestinal destruction of the upper part of the epithelium with infiltration by inflammatory cells. Rab or Von treatment aggravated the histological damage with further necrotic change of the epithelium and inflammatory cell infiltration compared to vehicle-treated group. The scale bar is 200 μm. (**F**,**G**) The mRNA expression levels of interleukin-1β (IL-1β) (**F**) and tumor necrosis factor-α (TNF-α) (**G**). mRNA levels are expressed as ratios to the mean value for normal small intestinal tissue. Each column represents mean ± SEM, N = 7. **P < 0.01, *P < 0.05 vs. vehicle-treated controls.

Macroscopic small intestinal damage, which was revealed by dark blue staining with 1% Evans blue, was observed after IND administration (Fig. 2A,B). Histologically, IND caused intestinal sloughing and destruction, which extended into the submucosal layer with massive infiltration of inflammatory cells (Fig. 2C).

Rabeprazole and vonoprazan administration at high doses markedly elevated the lesion indices (Fig. 2B), and histologically, widened the area of IND-induced tissue injury (Fig. 2D,E) by increasing the mRNA expression level of IL-1β (Fig. 2F) and TNF-α (Fig. 2G) in the small intestine. Treatment with rabeprazole or vonoprazan alone did not change the macroscopic and microscopic findings (Supplemental Fig. 2A–C) in the small intestine and changes in the expression of IL-1β (Supplemental Fig. 2D) and TNF-α mRNA (Supplemental Fig. 2E) were not observed.

### IND-induced small intestinal injury worsens by transplanting small intestinal contents from rabeprazole- and vonoprazan-administered mice

**Figure 3:**
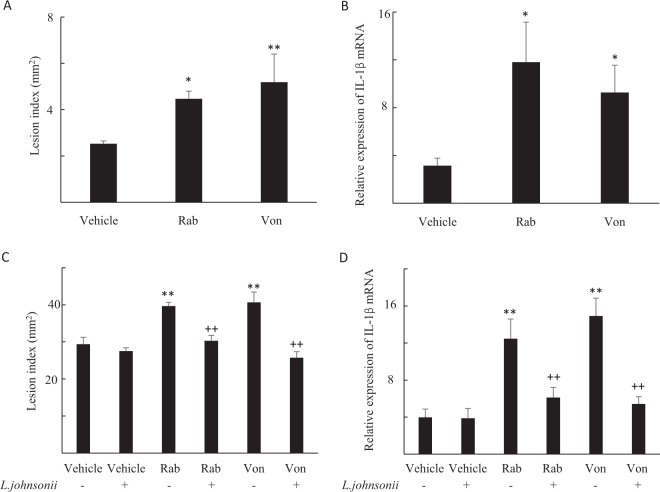
Small intestinal damage and cytokine expression after transplanting the fecal microbiota or supplementation of *Lactobacillus jhonsonii*. (**A**,**B**) Small intestinal damage and cytokine expression after transplanting the fecal microbiota of small intestinal luminal contents from the vehicle-, rabeprazole (Rab)- or vonoprazan (Von)-treated mice. (**A**) The areas of the macroscopically visible lesions were measured, summed per small intestine, and used as the lesion index. Each column represents mean ± standard error of the mean (SEM). **P < 0.01, *P < 0.05 vs. vehicle-treated controls, N = 4–8. (**B**) The mRNA expression levels of interleukin-1β (IL-1β). mRNA levels are expressed as ratios to the mean value for normal small intestinal tissue. Each column represents mean ± SEM, N = 5–6. **P < 0.01, *P < 0.05 vs. vehicle-treated controls. (**C**,**D**) Small intestinal damage and cytokine expression with or without *Lactobacillus johnsonii* (*L. johnsonii*) in the vehicle-, Rab- or Von-treated mice. (**C**) The areas of the macroscopically visible lesions were measured, summed per small intestine, and used as the lesion index. Each column represents mean ± standard error of the mean (SEM). **P < 0.01 vs. vehicle-treated controls, ^++^P < 0.01 vs. corresponding group without *L. johnsonii* treatment, N = 6–8. (**D**) The mRNA expression levels of IL-1β mRNA levels are expressed as ratios to the mean value for normal small intestinal tissue. Each column represents mean ± SEM, N = 5–8. **P < 0.01 vs. vehicle-treated controls, ^++^P < 0.01 vs. corresponding group without *L. johnsonii* treatment.

Recipient mice transplanted with small intestinal contents from rabeprazole and vonoprazan- treated mice experienced aggravated small intestinal injury (Fig. 3A), and elevated IL-1β mRNA expression after IND treatment (Fig. 3B).

### *Lactobacillus johnsonii* supplementation ameliorates IND-induced small intestinal injury after rabeprazole and vonoprazan administration

The administration of *L. johnsonii* ameliorated IND-induced small intestinal injury at doses ≥10^6^, while *L. johnsonii* at a dose of 10^5^ did not alter the injury (Supplemental Fig. 3A,B). The administration of *L. johnsonii* at a dose of 10^5^ prevented the increases in lesion indices and IL-1β mRNA expression 24 h after IND challenge to mice treated with rabeprazole and vonoprazan (Fig. 3C,D).

### *L*. murinus but not *L. intestinalis* ameliorates IND-induced small intestinal injury

The effect of *L. murinus* and *L. intestinalis* on IND-induced small intestinal injury was also examined. The administration of *L. murinus* ameliorated IND-induced small intestinal injury at doses of 10^5^ and 10^6^, whereas *L. intestinalis* at any doses did not alter injury (Supplemental Fig. 3C,D).

### The administration of exogenous gastrin does not alter the severity of IND-induced small intestinal injury

We finally examined the effect of hypergastrinemia on IND-induced small intestinal injury. The administration of gastrin at 1 or 10 μg/mouse did not alter lesion indices (Supplemental Fig. 4A) or *IL-1β* mRNA expression (Supplemental Fig. 4B) 24 h after IND challenge.

## Discussion

The present study demonstrated that rabeprazole and vonoprazan administration altered the small intestinal microbiome, and resulted in high susceptibility to IND-induced small intestinal injury in mice. Since this phenotype was mimicked by transplanting small intestinal contents from mice given rabeprazole or vonoprazan, these alterations in the small intestinal microbiome serve as the key complicating factor of IND-induced small intestinal injury. Our results also suggest that the decrease in *L. johnsonii* proportion in the small intestinal microbiome may be responsible for the worsening of IND-induced small intestinal injury after rabeprazole or vonoprazan administration.

Rabeprazole, one of the PPIs, and vonoprazan, one of the P-CABs, are both strong acid suppressors; however, they have a different mechanism of action. The present β-diversity results showed that the effect on the small intestinal microbiome also slightly differed. However, the dynamics of microbes after rabeprazole- and vonoprazan-treatment were similar in both groups, suggesting that this intestinal microbiome alteration was due to the gastric acid suppressing effect of the drugs, rather than the action of the drugs themselves.

In the present study, the decreased population of *L. johnsonii* in the small intestine was considered to be one of the causes of the deterioration in IND-induced small intestinal injury due to rabeprazole or vonoprazan administration because *L. johnsonii* supplementation to rabeprazole- or vonoprazan-treated mice terminated the aggravation of IND-induced small intestinal injury. *L. johnsonii* is often used as a probiotic owing to its wide-spectrum antibacterial activity^21^. In the murine intestinal *Campylobacter jejuni* infection model, *L. johnsonii* attenuated the intestinal mucosa and systemic pro-inflammatory immune response^22^. In our previous study, lactic acid, which is a representative product of *L. johnsonii* played protective roles in NSAID-induced small intestinal injury^12^. Consistent with these previous studies, *L. johnsonii* may play a protective role by itself or by producing lactic acid as shown in this study. The population of *L. johnsonii* in the small intestine was reduced following rabeprazole or vonoprazan administration, which may have been because *L. johnsonii* is an acid-resistant Lactobacillus, and the population ratio may decrease when other acid-susceptible bacteria increase by gastric acid suppression. Interestingly, *L. johnsonii* in the cecum contents did not change by rabeprazole and vonoprazan administration, which may be due to the small distance from the stomach; a downstream effect of acid suppression is stronger in the small intestine than in the colon.

Both *L. murinus* and *L. intestinalis* belong to the same genus as *L. Johnsonii*. However, contrary to that observed with *L. Johnsonii*, the population ratios of *L. murinus* and *L. intestinalis* were increased or tended to be increased with the administration of rabeprazole or vonoprazan. Our results demonstrated that the exogeneous administration of *L. intestinalis* affected neither IND-induced small intestinal injury, as assessed by the lesion index, nor the mRNA expression of *IL-1β*, suggesting that this alteration might not play a significant role in IND-induced small intestinal injury. In contrast, the exogeneous administration of *L. murinus* ameliorated IND-induced small intestinal injury. From this result, it is assumed that *L. murinus* might play a protective role in NSAID-induced small intestinal injury. However, the increase in *L. murinus* by these drugs did not lead to the inhibition of IND-induced injury. Since both rabeprazole and vonoprazan decreased the abundance ratio of *L. johnsonii*, which is the most predominant species in the small intestine and has an inhibitory effect on damage, the increased protective effect of *L. murinus* associated with gastric acid inhibition seems to be abolished by the reduction of *L. johnsonii*, resulting in aggravation of IND-induced small intestinal injury.

An earlier study proposed the possible involvement of Actinobacteria in the exacerbation of NSAID-induced small intestinal injury associated with PPI treatment. Wallace *et al*.^11^ demonstrated that the number of Actinobacteria was significantly reduced in the jejunum by omeprazole treatment, but was not altered in the colon. Similarly, we observed a reduction in the population of Actinobacteria in the small intestine after rabeprazole and vonoprazan administration, although our analysis did not detect any species responsible for this reduction. Considering the small population of Actinobacteria in the small intestine, the contribution by the reduction of this bacteria, if any, might be much smaller than that of the reduction of *L. johnsonii* in the exacerbation of NSAID-induced injury.

The long-term administration of PPIs is known to cause small intestinal bacterial overgrowth in humans^23,24^. However, although the composition of small intestinal bacteria was changed, the total bacterial number, as measured based on the TUF gene, was not altered after rabeprazole and vonoprazan administration in the present study. The reason for this discrepancy is unclear, but it might be because the period of PPI administration was as short as 1 week in our experiments. As a specific bacterial alteration in the small intestine after gastric acid inhibitor administration, changes in OTUs and composition might occur first, followed by an increase in the number of bacteria. To elucidate the detailed mechanism, it is necessary to confirm this by examining the long-term administration of gastric acid inhibitors. However, in any case, our experimental results show that an increase in the number of bacteria is not essential for the worsening of injury, and a compositional change characterized by a decrease in *L. johnsonii* is important for the exacerbation of IND-induced small intestinal injury.

Recent human studies have reported similar alterations in the gut microbiome, with a significant change in β-diversity indices for PPI users; the detailed results however differed in each study^25^. Additionally, in most studies, PPI usage was associated with a higher abundance of *Lactobacillus*, and no study found changes in the population of *L. johnsonii* in such users^26–30^. It should be noted that these studies were performed using stool sample. Considering that unlike the small intestine, we failed to detect the decreases in *L. johnsonii* population in the colon of mice given rabeprazole and vonoprazan, site-specific alterations may occur after the administration of the acid suppressants.

Some studies have suggested that fecal samples cannot represent the entire microbiota in the digestive tract, and proper GI samples should be selected when diseases are discussed^31–34^. The colon receives less influence from transient microorganisms than small intestine, and offers a better surrounding for bacterial growth^34^. Additionally, since the effect of gastric acid suppression is stronger in the small intestine, alterations of the microbiome in the small intestine and the large intestine by vonoprazan or rabeprazole should not be considered the same, and hence, should be evaluated separately. Indeed, our results suggested that the alteration in α-diversity of the small intestine microbiome by vonoprazan or rabeprazole treatment was more prominent compared to that in the colon, and changes in the population of some species in the small intestine and colon were different. However, studies of human or animal small intestinal microbiome are rare because of the difficulty to collect small intestinal samples, and the overestimation of the significance of colonic or fecal microbiome in the pathophysiology of intestinal diseases. To our knowledge, this is the first report to focus on the influence of gastric acid suppression in small intestinal microbiome using the 16S rRNA sequencing method. Here, we identified the specific bacterial species, *L. johnsonii*, whose population was dramatically decreased in the small intestine, but not in the large intestine after treatment with acid inhibitors. We found that this reduction was the key factor associated with the high sensitivity to damage in gastric acid-suppressed mice.

In conclusion, our result suggested that rabeprazole and vonoprazan aggravated the NSAID-induced small intestinal injury via reducing the population of *L. johnsonii* in the small intestine which is associated with the suppression of gastric acid secretion. *L. johnsonii* supplementation therapy can therefore serve as a new treatment for NSAID-induced small intestinal injury. Microbiome studies with NGS focusing on the human small intestine, instead of the colon or feces, are urgently warranted to gain a better understanding of the pathophysiology of NSAID-induced small intestinal injury.

## Supplementary information

Supplemental documents

Supplemental Table 6

Supplemental Table 7

Supplemental Table 8
