# Supplementary material for: Gastric acid inhibitor aggravates indomethacin-induced small intestinal injury via reducing Lactobacillus johnsonii
Source: Sci Rep. 2019 Nov 25;9:17490. doi: 10.1038/s41598-019-53559-7 (PMC6877529; doi:10.1038/s41598-019-53559-7)
Supplement: Supplementary file 1 — Supplemental documents [file 41598_2019_53559_MOESM1_ESM.pdf]

Gastric acid inhibitor aggravates indomethacin-induced small intestinal injury via reducing *Lactobacillus johnsonii.*

**Short title:** PPI and P-cab aggravate NSAIDs-induced injury

Yuji Nadatani[1], Toshio Watanabe*[1], Wataru Suda[2], Akinobu Nakata[1], Yuji Matsumoto[1], Satoshi Kosaka[1], Akira Higashimori[1], Koji Otani[1], Shuhei Hosomi[1], Fumio Tanaka[1], Yasuaki Nagami[1], Noriko Kamata[1], Koichi Taira[1], Hirokazu Yamagami[1], Tetsuya Tanigawa[1], Masahira Hattori[2], Yasuhiro Fujiwara[1]

1.Department of Gastroenterology, Osaka City University Graduate School of Medicine, Osaka, Japan

2.Laboratory for Microbiome Sciences, Center for Integrative Medical Sciences, RIKEN, Yokohama, Kanagawa, Japan.

Supplemental Figure 1.

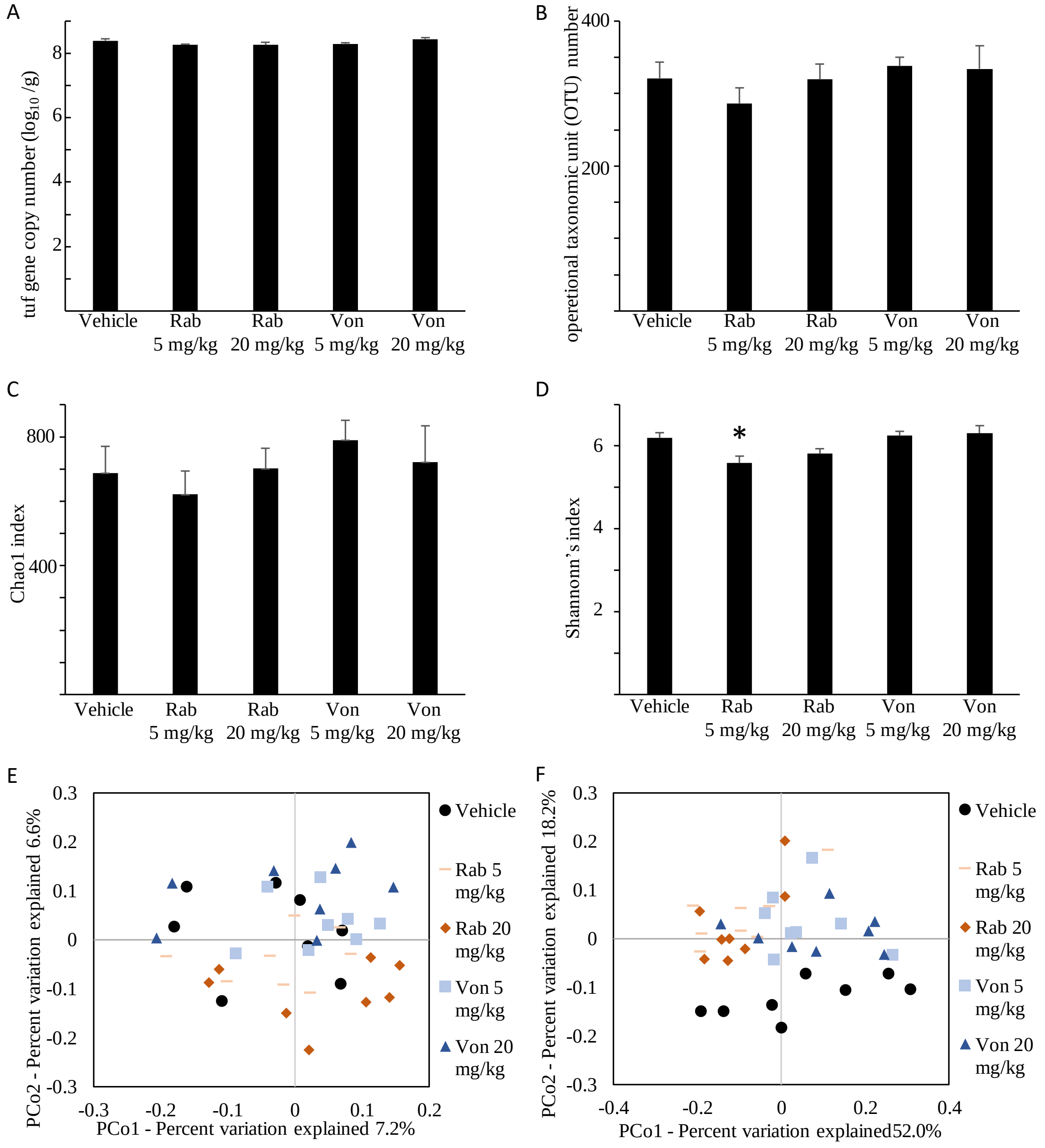

Supplemental Figure 1. Changes in the colon microbiome following administration of rabeprazole and vonoprazan. (A) Total number of a wide variety of bacterial strains in the colon of mice administered rabeprazole (Rab), vonoprazan (Von) or vehicle. Total number of bacterial strains were calculated based on the tuf gene copy number per gram of luminal contents of small intestine; N = 7-8. (B-D) Alpha-diversity, measured by Operational taxonomic unit (OTU) number (B), Shannon's diversity index (C), and Chao1 index (D) of small intestinal lumen contents in mice given Rab, Von or vehicle; N=7-8. *$p < 0.05$ compared to the OTU number in vehicle-treated mice. (E, F) Principal coordinate analysis (PCoA) of unweighted (E) and weighted (F) UniFrac distances of small intestinal lumen contents.

Supplemental Figure 2.

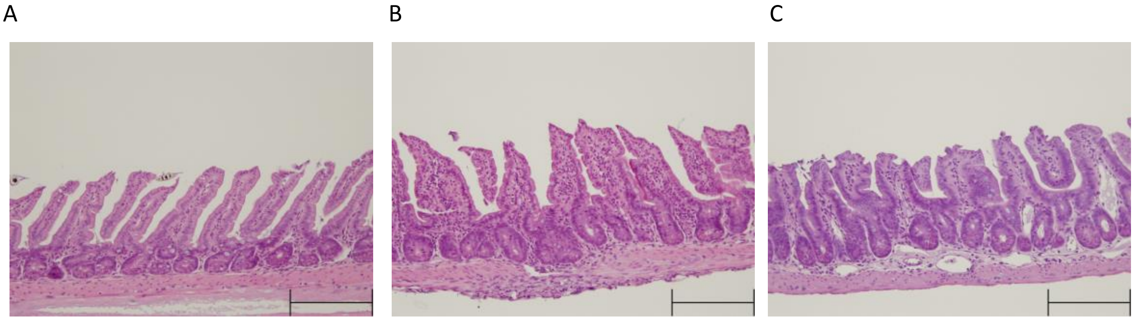


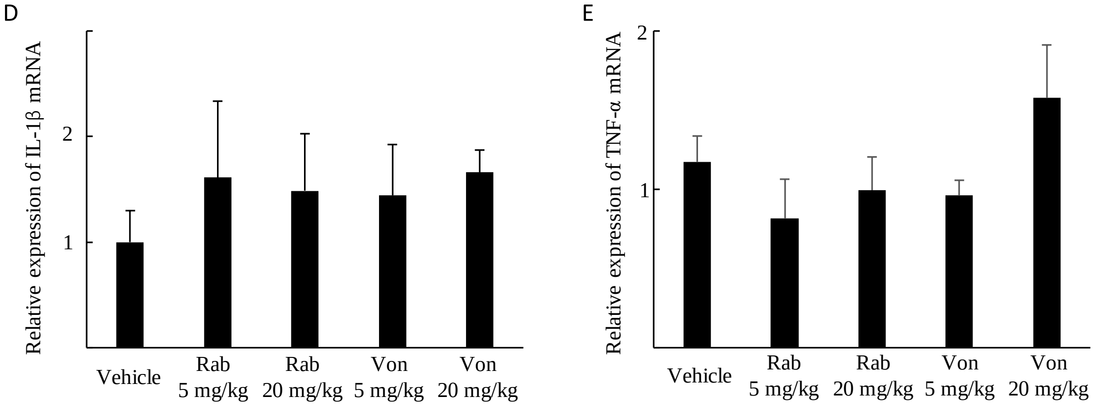

Supplemental Figure 2. Histological changes in colon and cytokine expression following indomethacin administration to rabeprazole-, vonoprazan- and vehicle-treated mice. (A-C) Histological findings of indomethacin-induced injury in the colon of the vehicle- (A), Rab- (B) or Von- (C) treated groups. Indomethacin, and Rab or Von treatment did not affect the colon histologically. The scale bar is 200 μm. (D, E) The mRNA expression levels of interleukin-1β (IL-1β) (D) and tumor necrosis factor-α (TNF-α) (E); mRNA levels are expressed as ratios to the mean value for normal small intestinal tissue. Each column represents mean ± SEM (N = 7). $^{**}P < 0.01$, $^{*}P < 0.05$ vs. vehicle-treated controls.

Supplemental Figure 3.

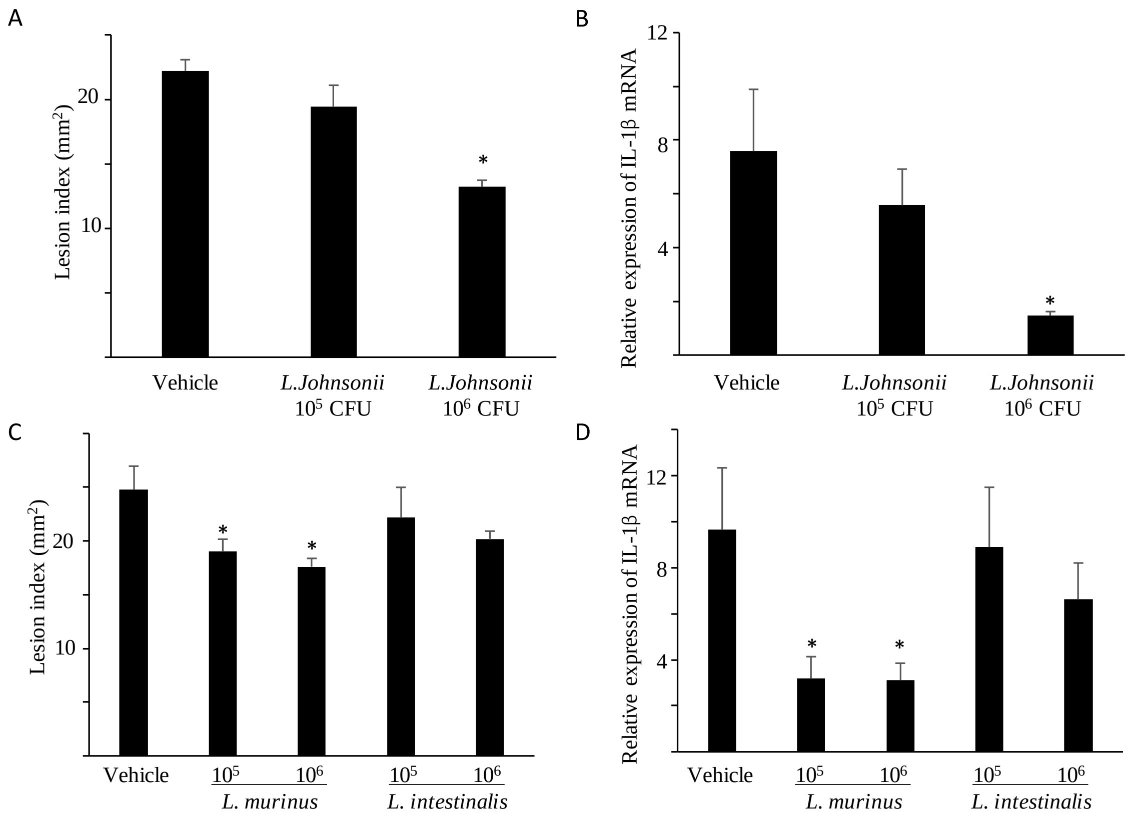

Supplemental Figure 3. Small intestinal damage and cytokine expression after the administration of *Lactobacillus johnsonii*, *Lactobacillus murinus*, and *Lactobacillus intestinalis*. (A, B) Small intestinal damage and cytokine expression with or without $10^5$ and $10^6$ CFU *L, johnsonii*. (A) The areas of macroscopically visible lesions were measured, assessed per small intestine, and used as the lesion index. Each column represents the mean ± standard error of the mean (SEM). *P < 0.05 vs. vehicle-treated controls. N = 5. (B) The mRNA expression levels of *IL-1β* are expressed as ratios relative to the mean value for normal small intestinal tissue. Each column represents mean ± SEM, N = 5–7. *P < 0.05 vs. vehicle-treated controls. (C, D) Small intestinal damage and cytokine expression with or without $10^5$ and $10^6$ CFU *L. murinus* or *L. intestinalis*. (C) The areas of macroscopically visible lesions were measured, assessed per small intestine, and used as the lesion index. Each column represents the mean ± SEM. *P < 0.05 vs. vehicle-treated controls. N = 5. (D) The mRNA expression levels of *IL-1β* are expressed as ratios relative to the mean value for normal small intestinal tissue. Each column represents mean ± SEM, N = 5–7. *P < 0.05 vs. vehicle-treated controls.

Supplemental Figure 4.

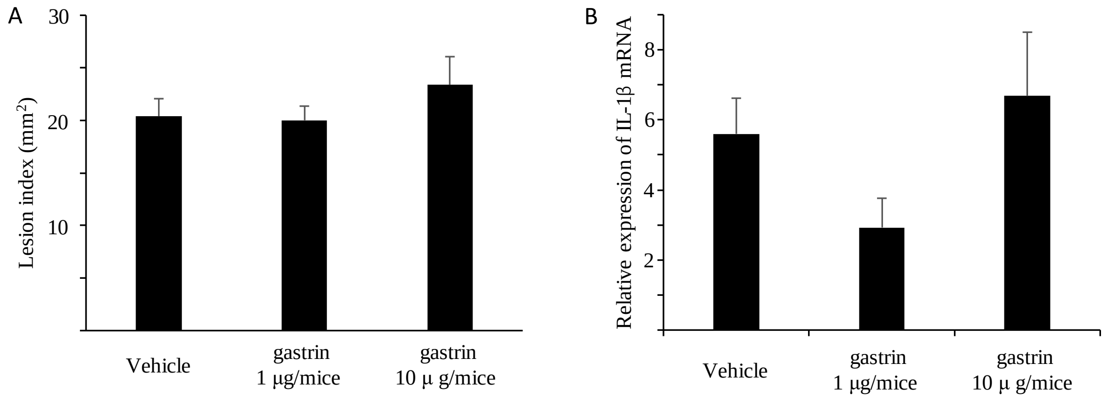

Supplemental Figure 4. Small intestinal damage and cytokine expression after the administration of gastrin at a dose of 1 μg or 10 μg/mouse. (A, B) Small intestinal damage and cytokine expression with or without the administration of gastrin (A). The areas of macroscopically visible lesions were measured, assessed per small intestine, and used as the lesion index. Each column represents the mean ± standard error of the mean (SEM). N = 5. (B) The mRNA expression levels of *IL-1β* are expressed as ratios relative to the mean value for normal small intestinal tissue. Each column represents the mean ± SEM, N = 5–7.

Supplemental Table 1. Primers and Probes

| Gene | | Primer and Probe |
|---|---|---|
| TNF-α | Primer (forward) | 5’-TCATGCACCACCATCAAGGA-3’ |
| | Primer (reverse) | 5’-GAGGCAACCTGACCACTCTCC-3’ |
| | Probe | 5’-FAM-AATGGGCTTTCCGAATTCACTGGAGC-TAMRA-3’ |
| IL-1β | Primer (forward) | 5’-ACAGGCTCCGAGATGAACAAC-3’ |
| | Primer (reverse) | 5’-CCATTGAGGTGGAGAGCTTTC-3’ |
| | Probe | 5’-FAM-GAAAAAGCCTCGTGCTGTCGGACCCATAT-TAMRA-3’ |

Supplemental Table 2. The major bacterial compostion of colon at phylum level. (%)

| | vehicle | rabeprazole | | vonoprazan | |
|---|---|---|---|---|---|
| | | 5 mg/kg | 20 mg/kg | 5 mg/kg | 20 mg/kg |
| Firmicutes | 65.52 ± 4.8279 | 61.41 ± 3.9327 | 60.43 ± 3.325 | 73.12 ± 2.2709 | 73.671 ± 3.11 |
| Bacteroidetes | 31.87 ± 4.4111 | 34.03 ± 3.539 | 35.31 ± 3.1827 | 24.43 ± 1.9282 | 23.567 ± 2.89 |
| Verrucomicrobia | 1.92 ± 0.4774 | 3.62 ± 0.6356 * | 3.40 ± 0.4208 * | 1.72 ± 0.474 | 2.1625 ± 0.45 |
| Actinobacteria | 0.64 ± 0.1303 | 0.90 ± 0.0935 | 0.85 ± 0.1424 | 0.63 ± 0.1192 | 0.5375 ± 0.07 |
| Proteobacteria | 0.00 ± 0 | 0.03 ± 0.016 | 0.00 ± 0.0042 | 0.05 ± 0.0259 * | 0.0333 ± 0.02 |
| Deferribacteres | 0.03 ± 0.0248 | 0.00 ± 0 | 0.00 ± 0 | 0.02 ± 0.014 | 0.0167 ± 0.01 |
| Tenericutes | 0.02 ± 0.0088 | 0.01 ± 0.0055 | 0.02 ± 0.0063 | 0.02 ± 0.0088 | 0.0125 ± 0.01 |

Identity ≥ 70% Mean ± s.e. *; $p < 0.05$ vs Vehicle

Supplemental Table 3 The major bacterial compostion of colon at genus level. (%)

| | vehicle | rabeprazole | | | | vonoprazan | | | |
|---|---|---|---|---|---|---|---|---|---|
| | | 5 mg/kg | | 20 mg/kg | | 5 mg/kg | | 20 mg/kg | |
| Lactobacillus | 9.00 ± 1.78 | 28.38 ± 1.94 | ** | 24.63 ± 2.12 | ** | 21.11 ± 2.96 | ** | 17.68 ± 2.15 | ** |
| Clostridium | 35.99 ± 5.37 | 18.95 ± 3.16 | ** | 19.38 ± 2.26 | ** | 31.76 ± 2.90 | | 33.20 ± 2.65 | |
| Akkermansia | 1.90 ± 0.48 | 3.59 ± 0.64 | ** | 3.35 ± 0.42 | ** | 1.70 ± 0.46 | | 2.12 ± 0.44 | |
| Eubacterium | 2.68 ± 0.32 | 2.36 ± 0.28 | | 1.55 ± 0.18 | * | 2.15 ± 0.27 | | 2.49 ± 0.56 | |
| Roseburia | 1.12 ± 0.22 | 0.96 ± 0.25 | | 0.68 ± 0.20 | | 2.18 ± 0.40 | * | 2.01 ± 0.58 | |
| Candidatus Arthromitus | 0.11 ± 0.02 | 0.20 ± 0.08 | | 0.05 ± 0.02 | | 0.16 ± 0.05 | | 0.07 ± 0.03 | |
| Ruminococcus | 0.50 ± 0.10 | 0.70 ± 0.21 | | 1.90 ± 0.38 | ** | 1.12 ± 0.38 | | 1.36 ± 0.53 | |
| Oscillospira | 0.68 ± 0.13 | 0.55 ± 0.11 | | 0.22 ± 0.04 | * | 0.52 ± 0.16 | | 0.95 ± 0.24 | |
| Parabacteroides | 0.54 ± 0.08 | 0.43 ± 0.10 | | 0.65 ± 0.10 | | 0.52 ± 0.10 | | 0.51 ± 0.11 | |
| Escherichia | 0.00 ± 0.00 | 0.02 ± 0.02 | | 0.00 ± 0.00 | | 0.05 ± 0.03 | * | 0.02 ± 0.02 | |

Identity ≥ 94% Mean ± s.e. *; $p < 0.05$ vs Vehicle, **; $p < 0.01$ vs Vehicle

Supplemental Table 4 The major bacterial composition of colon at species level. (%)

| | | | vehicle | rabeprazole | | | | vonoprazan | | | |
|---|---|---|---|---|---|---|---|---|---|---|---|
| | strain | % id | | 5 mg/kg | | 20 mg/kg | | 5 mg/kg | | 20 mg/kg | |
| total_OTU00007 | Lactobacillus johnsonii | 100.0 | 3.35 ± 0.57 | 7.18 ± 0.92 | ** | 5.24 ± 0.68 | | 4.43 ± 0.59 | | 4.53 ± 0.75 | |
| total_OTU00029 | Barnesiella intestinihominis | 86.5 | 9.23 ± 1.12 | 10.52 ± 0.87 | | 10.33 ± 0.78 | | 7.72 ± 0.53 | | 8.08 ± 1.01 | |
| total_OTU00016 | Lactobacillus intestinalis | 100.0 | 1.25 ± 0.34 | 7.56 ± 0.75 | ** | 8.38 ± 0.68 | ** | 8.22 ± 1.20 | ** | 5.92 ± 0.85 | ** |
| total_OTU00013 | Lactobacillus reuteri | 100.0 | 2.14 ± 0.48 | 7.93 ± 1.32 | ** | 6.89 ± 0.77 | ** | 4.33 ± 0.55 | | 3.69 ± 0.36 | |
| total_OTU00041 | Barnesiella intestinihominis | 88.6 | 4.17 ± 0.54 | 4.48 ± 0.63 | | 4.09 ± 0.28 | | 3.26 ± 0.37 | | 2.60 ± 0.32 | * |
| total_OTU00024 | Lactobacillus murinus | 100.0 | 2.03 ± 0.71 | 4.94 ± 0.73 | ** | 3.23 ± 0.67 | | 3.45 ± 0.97 | | 3.06 ± 0.42 | |
| total_OTU00096 | Barnesiella intestinihominis | 86.9 | 2.71 ± 0.46 | 3.19 ± 0.35 | | 3.64 ± 0.43 | | 1.71 ± 0.16 | * | 1.85 ± 0.18 | |
| total_OTU00129 | Porphyromonas HF001 | 86.0 | 2.75 ± 0.46 | 3.58 ± 0.51 | | 2.53 ± 0.38 | | 1.90 ± 0.25 | | 2.31 ± 0.39 | |
| total_OTU00116 | Porphyromonas catoniae | 88.1 | 3.05 ± 0.40 | 2.75 ± 0.41 | | 3.91 ± 0.46 | | 2.84 ± 0.20 | | 2.31 ± 0.30 | |
| total_OTU00097 | Porphyromonas catoniae | 86.0 | 2.08 ± 0.30 | 1.95 ± 0.32 | | 1.77 ± 0.29 | | 1.94 ± 0.22 | | 1.38 ± 0.20 | |
| total_OTU00188 | Barnesiella intestinihominis | 87.2 | 1.18 ± 0.27 | 1.61 ± 0.32 | | 1.56 ± 0.23 | | 0.68 ± 0.12 | | 0.82 ± 0.16 | |
| total_OTU00546 | Porphyromonas catoniae | 86.9 | 1.78 ± 0.40 | 1.08 ± 0.19 | * | 1.22 ± 0.14 | | 0.79 ± 0.13 | ** | 0.71 ± 0.16 | ** |
| total_OTU00540 | Porphyromonas catoniae ATCC 51270 | 85.0 | 1.11 ± 0.27 | 1.55 ± 0.24 | | 1.67 ± 0.22 | | 0.68 ± 0.09 | | 0.66 ± 0.15 | |
| total_OTU00017 | Candidatus Arthromitus sp. SFB-mouse | 100.0 | 0.11 ± 0.02 | 0.19 ± 0.08 | | 0.05 ± 0.02 | | 0.15 ± 0.05 | | 0.07 ± 0.03 | |
| total_OTU00208 | Akkermansia muciniphila | 100.0 | 1.88 ± 0.48 | 3.57 ± 0.63 | * | 3.33 ± 0.42 | * | 1.70 ± 0.46 | | 2.09 ± 0.44 | |
| total_OTU00207 | Bacteroides sp. Tilapia9 | 84.1 | 0.98 ± 0.14 | 1.09 ± 0.17 | | 1.29 ± 0.20 | | 0.78 ± 0.08 | | 0.94 ± 0.18 | |
| total_OTU00127 | Clostridium disporicum | 99.5 | 0.69 ± 0.19 | 0.05 ± 0.01 | * | 0.02 ± 0.01 | ** | 0.15 ± 0.09 | * | 0.97 ± 0.31 | |
| total_OTU00276 | Eubacterium biforme DSM 3989 | 87.3 | 0.40 ± 0.21 | 0.68 ± 0.19 | | 3.10 ± 0.42 | ** | 0.02 ± 0.01 | | 0.15 ± 0.07 | |
| total_OTU00399 | Escherichia coli | 100.0 | 0.00 ± 0.00 | 0.02 ± 0.02 | | 0.00 ± 0.00 | | 0.05 ± 0.03 | | 0.02 ± 0.02 | |
| total_OTU00143 | [Bacillus] sp. KITNT-3 | 100.0 | 0.02 ± 0.01 | 0.02 ± 0.01 | | 0.00 ± 0.00 | | 0.21 ± 0.12 | * | 0.07 ± 0.03 | |

Mean ± s.e. *; $p < 0.05$ vs Vehicle, **; $p < 0.01$ vs Vehicle

Supplemental Table 5. ANOSIM of unifrac distance in colon

| Category | No. subject | Weigthed UniFrac | | Unweigthed UniFrac | |
|---|---|---|---|---|---|
| | | $R^2$ | P value | $R^2$ | P value |
| vehicle vs rabeprazole 5mg/kg | vehicle:8<br>rabeprazole 5mg/kg:8 | 0.34 | <0.01 | 0.09 | <0.01 |
| vehicle vs rabeprazole 20mg/kg | vehicle:8<br>rabeprazole 20mg/kg:8 | 0.30 | 0.01 | 0.11 | <0.01 |
| vehicle vs vonoprazan 5mg/kg | vehicle:8<br>vonoprazan 5mg/kg:8 | 0.16 | 0.06 | 0.09 | <0.01 |
| vehicle vs vonoprazan 20mg/kg | vehicle:8<br>vonoprazan 20mg/kg:7 | 0.16 | 0.05 | 0.08 | 0.04 |
| rabeprazole 5mg/kg vs vonoprazan 5mg/kg | rabeprazole 5mg/kg:8<br>vonoprazan 5mg/kg:8 | 0.27 | 0.01 | 0.09 | <0.01 |
| rabeprazole 20mg/kg vs vonoprazan 20mg/kg | rabeprazole 20mg/kg:8<br>vonoprazan 20mg/kg:7 | 0.35 | <0.01 | 0.11 | <0.01 |
